# Oxygen conditions oscillating between hypoxia and hyperoxia induce different effects in the pulmonary endothelium compared to constant oxygen conditions

**DOI:** 10.14814/phy2.14590

**Published:** 2021-02-10

**Authors:** Peter Wohlrab, Michael Johann Danhofer, Wolfgang Schaubmayr, Akos Tiboldi, Katharina Krenn, Klaus Markstaller, Roman Ullrich, Klaus Ulrich Klein, Verena Tretter

**Affiliations:** ^1^ Department of Anesthesia and General Intensive Care Medical University Vienna Vienna Austria

**Keywords:** hyperoxia, intermittent hyperoxia, lung microvascular endothelial cells, oxygen oscillations

## Abstract

The pulmonary endothelium is an immediate recipient of high oxygen concentrations upon oxygen therapy and mediates down‐stream responses. Cyclic collapse and reopening of atelectatic lung areas during mechanical ventilation with high fractions of inspired oxygen result in the propagation of oxygen oscillations in the hypoxic/hyperoxic range. We used primary murine lung endothelial cell cultures to investigate cell responses to constant and oscillating oxygen conditions in the hypoxic to hyperoxic range. Severe constant hyperoxia had pro‐inflammatory and cytotoxic effects including an increase in expression of ICAM1, E‐selectin, and RAGE at 24 hr exposure. The coagulative/fibrinolytic system responded by upregulation of uPA, tPA, and vWF and PAI1 under constant severe hyperoxia. Among antioxidant enzymes, the upregulation of SOD2, TXN1, TXNRD3, GPX1, and Gstp1 at 24 hr, but downregulation of SOD3 at 72 hr constant hyperoxia was evident. Hypoxic/hyperoxic oscillating oxygen conditions induced pro‐inflammatory cytokine release to a lesser extent and later than constant hyperoxia. Gene expression analyses showed upregulation of NFKB p65 mRNA at 72 hr. More evident was a biphasic response of NOS3 and ACE1 gene expression (downregulation until 24 hr and upregulation at 72 hr). ACE2 mRNA was upregulated until 72 hr, but shedding of the mature protein from the cell surface favored ACE1. Oscillations resulted in severe production of peroxynitrite, but apart from upregulation of Gstp1 at 24 hr responses of antioxidative proteins were less pronounced than under constant hyperoxia. Oscillating oxygen in the hypoxic/hyperoxic range has a characteristical impact on vasoactive mediators like NOS3 and on the activation of the renin‐angiotensin system in the lung endothelium.

## Graphical abstract

Hypoxic/hyperoxic oscillating oxygen conditions is shown to induce pro‐inflammatory cytokine release to a lesser extent and later than constant hyperoxia. Evident was severe production of peroxynitrite, up‐regulation of NFKB p65 mRNA at 72 hr, a biphasic response of TNFa, NOS3, and ACE1 gene expression (down‐regulation until 24 hr and up‐regulation at 72 hr) and up regulation of NFKB at 72 hr. ACE2 mRNA was continuously up‐regulated until 72 hr, but shedding of the mature protein from the cell surface seemed to favor ACE1.

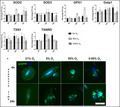

## 1 INTRODUCTION

Oxygen is vital for higher organisms, and organ dysfunctions frequently lead to an oxygen deficit and concurrent hypoxemia that requires treatment with supplemental oxygen eventually together with mechanical ventilation. On the other side of the scale, oxygen toxicity is encountered, a term which describes pathological changes due to excessive hyperoxic exposure, with the lung being the primarily affected organ. Examples are conditions like bronchopulmonary dysplasia and hyperoxic acute lung injury (HALI) (Ali, Schmidt, Dodd, & Jeppesen, 2013; Kallet & Matthay, 2013). The lung and especially its endothelium are targets that experience the highest oxygen concentrations in the body and are also first responders. High fractions of inspired oxygen (FiO_2_), for example, in the perioperative period, lead to absorption atelectasis also in the healthy lung. Cyclic recruitment and de‐recruitment in the already injured lung can result in oscillations of partial pressure of oxygen that have been shown to be transmitted to distant tissues in animal experiments (Klein, Boehme, et al., 2013; Klein, Hartmann, et al., 2013). Oxygen oscillations in the hypoxic range (commonly termed intermittent hypoxia) have attracted broad interest as the hallmark of conditions such as obstructive sleep apnea. Molecular mechanisms of intermittent hypoxia have been widely investigated and have been shown to induce signaling pathways which differ from constant oxygen conditions. Oscillating oxygen conditions have repeatedly been shown to induce pathological developments and injury to various organs (Boehme et al., 2019; Drager et al., 2013; Klein et al., 2016; Prabhakar & Semenza, 2012). In this study, we addressed changes of gene expression in murine lung endothelium in response to combined intermittent hypoxia/hyperoxia, as compared to constant hypoxic, normoxic, and hyperoxic conditions. The observed alterations in molecular patterns induced by different oxygen conditions affected important aspects of endothelial cell function including cytokine release, expression of cell adhesion molecules, regulation of coagulation, fibrinolysis, and vascular tone as well as redox systems.

## 2 MATERIALS AND METHODS

### 2.1 Ethics committee approval

Primary murine lung endothelial cells were derived from adult C57B/L6 WT mice. Approval for the use of mice was obtained after a hearing in front of the Medical University Vienna animal experimentation ethics committee by the Federal Ministry of education, science and research (BMBWF‐66.009/0136‐V/3b/2018). Animals were obtained from the Center for Biomedical Research, a department that has implemented the ARRIVE guidelines.

### 2.2 Isolation of Murine Lung Endothelial Cells (MLECs)

Mouse lung endothelial cells were isolated and purified by magnetic separation as described in (Zirlik et al., 2007). The cells were seeded on plates coated with gelatine containing 10 μg/ml fibronectin (Sigma‐Aldrich). Plating medium was M199/Glutamax medium (ThermoFisher), 20% fetal calf serum superior (Biochrom), 30 μg/ml endothelial growth supplement from bovine pituitary (Sigma‐Aldrich), 5 U/ml heparin (Sigma), and antibiotics (penicillin/streptomycin/amphotericin; ThermoFisher). CD31 is a popular endothelial marker used to isolate this cell type and occurs only in low expression levels also in macrophages. This cell preparation results in a mixed population of endothelial cells from microvascular origin or larger vessels. Further purification would be possible using *Ulex Europaeus* lectin, which selects for microvascular endothelial cells, but this was omitted in this study, as it would decrease the yield significantly due to further manipulation and it was assumed, that the majority of cells numbers are of microvascular origin.

### 2.3 Exposure of cells to oxygen conditions

Three weeks after the isolation of MLECs, the cells were used for experiments. The cells were trypsinized and transferred into six‐well plates with gas‐permeable membranes (imaging plates: Zellkontakt). After 24 hr, the adherent cells were set up into custom‐made boxes, as described in Hafner et al. (2016), and exposed to four different oxygen conditions. These conditions were achieved by premixed gas bottles: (a) 21% O_2_‐5% CO_2_‐74% N_2_; (b) 5% O_2_‐ 5% CO_2_‐90% N_2_; (c) 95% O_2_‐5% CO_2_; (d) 0%–95% O_2_ oscillations‐ 5% CO_2_‐ rest N_2_. The oscillations were performed at a frequency of six oscillations per hour, as described in Wu et al. (2016). The analysis of cell responses was performed after 4, 24, and 72 hr.

### 2.4 Analysis of LDH and cytokine release

Cytotoxicity was addressed by lactate dehydrogenase (LDH) activity assay (Roche Diagnostics, Switzerland) using the supernatant of exposed MLECs. Cytokines released into supernatants were quantified by ELISA (Duoset; R&D Systems) using antibodies for KC/CXCL1, MIP2/CXCL2, and IL6.

### 2.5 Analysis of apoptotic/necrotic cells by flow cytometry

Cells were trypsinized and labeled using the FITC Annexin V Apoptosis Detection Kit (BD Pharmingen, BD Biosciences) to distinguish live, early and late apoptotic cells, as well as necrotic/dead cells by analysis on a FC 500 flow cytometer (Beckman Coulter, USA).

### 2.6 Analysis of gene expression and regulation of endothelial markers

Analysis of changes in gene expression was performed by quantitative real time PCR (qRT‐PCR). RNA was isolated using the RNeasy plus kit (Qiagen). Five hundred nanograms of mRNA were reverse transcribed using qScript cDNA synthesis kit (Quanta Biosciences). The resulting cDNA was analyzed on a RotorGene Q cycler (Qiagen) using PerfeCTa SYBR Green FastMix (Quanta Biosciences). For primer sequences see Table 1. The cycling program was 30 s 95°C for denaturing, followed by 45 cycles of 5 s 95°C, 15 s 56°C, and 10 s 72°C. Data analysis was performed by normalizing values to ß‐actin as a house‐keeping gene and comparing expression to normoxia (21% O_2_) exposure using the ΔΔCt method. Values generated by qPCR are given as “fold change compared to 21% O_2_.”

**TABLE 1:**
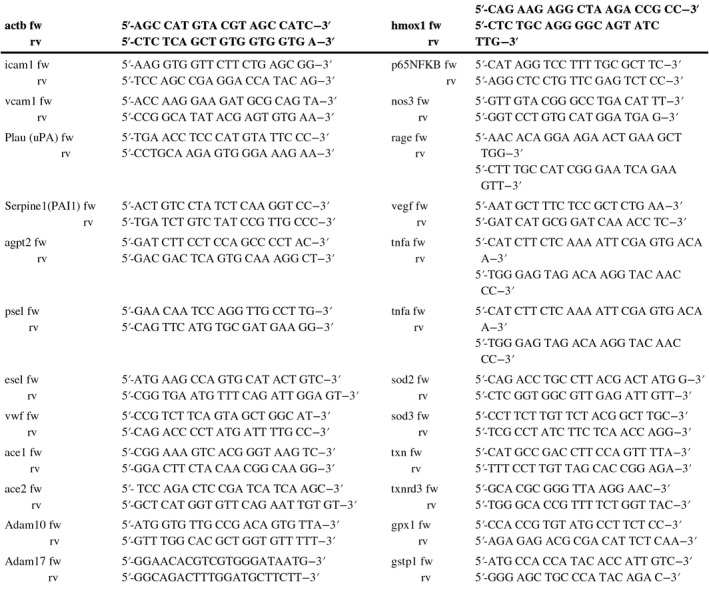
Nucleotide sequences of primers used in qPCR assays

Protein levels were analyzed by SDS‐PAGE and western blotting. Transferred proteins were detected by primary anti‐ACE/CD143 (R&D Systems cat.nr. AF1513), anti‐ACE2 (R&D Systems cat.nr.AF3437), anti‐eNOS (Cell Signaling Technology cat.nr. 9,586), anti‐ß‐Actin (Cell Signaling Technology cat.nr. 3,700) and according to species‐specific HRP‐coupled secondary antibodies (Rockland, USA). Bands were visualized by chemiluminescence (ECL Select Western blotting Detection Reagent; Amersham; GE Healthcare) using a Gel Documentation System (Vilber Lourmat).

### 2.7 Nitric oxide synthase activity assay

Nitric oxide (NO) is a gaseous radical with short physiological half‐life. Its generation can be quantified via its metabolites such as nitrite (NO_2_^−^) and nitrate (NO_3_^−^) (Bryan & Grisham, 2007). We used the Griess reagent kit (Abcam) to assess changes in NO metabolites in cell culture supernatants.

### 2.8 Live detection of peroxynitrite formation

We used a novel genetically encoded probe for detection of the subcellular formation of peroxynitrite, which is based on a boronic acid‐derived circularly permuted green fluorescent protein (pnGFP) and was designed in the laboratory of Hui‐wang Ai (University of California) (Chen, Ren, Wright, & Ai, 2013). For expression in MLECs, the plasmids pCDNA3‐pnGFP and pMAH‐POLY were transfected into MLECs using nucleofection (Amaxa/LONZA; Switzerland). 1.5 μg plasmid each were used to transfect 5 × 10^5^ cells. The transfected cells were put into culture and exposed to different oxygen conditions starting 24 hr later. After 4, 24, and 72 hr cells were either imaged live or after fixation with 4% para‐formaldehyde in PBS for 15 min at room temperature. Cell nuclei were highlighted by staining with DAPI. Imaging was performed on an Olympus IX83 Cell Vivo system.

### 2.9 Statistics

The experiments were conducted in at least six replicates and were repeated three times, each time with a new cell preparation. Mean values are given as mean ± standard deviation. Data in graphs depicted in the manuscript are from individual representative experiments with *n* = 6 replicates (=different cell culture wells, individually processed for analysis). Repeated experiments from new cell cultures gave comparable trends, but data were not pooled. We used GraphPad Prism (version7) for statistical analysis. As statistic tests were used for comparison of multiple groups at one time point: a one‐way anova, for more time points: a two‐way anova (Tukey's post‐hoc test); for comparing two groups (expression analysis: comparison to control condition‐21% O_2_): Student's *t*‐test. *p*‐values below .05 were regarded as statistically significant.

## 3 RESULTS

### 3.1 Constant hyperoxia and intermittent hypoxia/hyperoxia induce differential responses in the lung microvascular endothelium

#### 3.1.1 Cytokines and cellular viability

**FIGURE 1:**
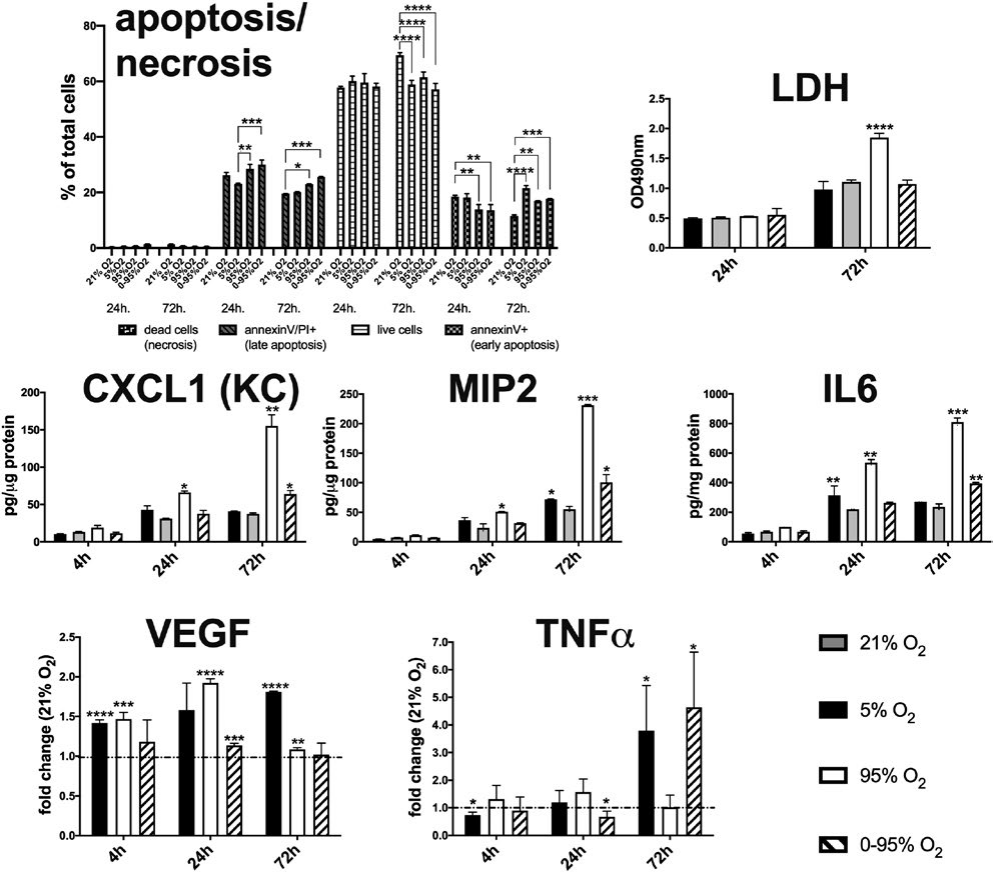
Cellular viability/cytotoxicity and cytokine release/expression in response to different oxygen conditions. (a) Flow cytometry analysis of apoptotic and necrotic cell states and lactate dehydrogenase (LDH) release analyzed by colorimetric test; (b) KC(CXCL1), MIP2 release into supernatant analyzed by ELISA. Cytokine (VEGF, TNFα) expression analyzed from cell lysate mRNA by qRT‐PCR. Results from flow cytometry, the colorimetric test and ELISA were analyzed by two‐way ANOVA with Tukey's multiple comparison test. Results from qRT‐PCR were analyzed using *actb* as house‐keeping gene and the ΔΔCt method (reference condition: 21% O_2_). For statistical testing a Student's *t*‐test was used: *p < .05; **p < .01; **p < .001;***p < .0001

We exposed murine lung endothelial cells to different hypoxic and hyperoxic oxygen conditions over a maximum duration of 72 hr, measuring cytotoxicity and release or expression of cytokines after 4, 24, and 72 hr by comparing read‐outs to 21% O_2_, which is regarded as the long‐term normoxic culturing condition. We used the combination of annexinV/propidium iodide staining to distinguish different apoptotic and necrotic cell stages (Figure 1a), and we observed a shift toward late apoptosis after 72 hr exposure to constant and oscillating hyperoxic conditions. Cell death might be underestimated by this method, as dead cells might deteriorate quickly and might escape detection by flow cytometry. LDH release is a surrogate measure of apoptotic/necrotic events accompanying cell death increasing to some extent during the normal culturing period. We observed a significant increase of LDH in the supernatant only under long‐term (72 hr) exposure to constant severe hyperoxia (95% O_2_), as compared to 21% O_2_ (Figure 1a). Similarly, cytokine release increased with culturing duration also under control conditions. However, release of KC (CXCL1), MIP2 (CXCL2), and IL6 was significantly elevated after 24 and 72 hr exposure to 95% O_2_ (KC: 66 ± 2 pg/μg protein (24 hr; *p* = .018) and 155 ± 14 pg/μg protein (72 hr; *p* = .007); MIP2: 51 ± 0.1 pg/μg protein (24 hr; *p* = .029) and 231 ± 1 pg/μg protein (72 hr; *p* = .0004); IL6: 540 ± 20 pg/mg protein (24 hr; *p* = .006) and 810 ± 28 pg/mg protein (72 hr; *p* = .0002); Figure 1a). Intermittent hypoxia/hyperoxia induced only a small, but still significant elevated cytokine release at the later time point (72 hr; KC: 64 ± 4 pg/μg protein; *p* = .016; MIP2: 100 ± 13 pg/μg protein; *p* = .044; IL6: 390 ± 6 pg/mg protein; *p* = .009; Figure 1a). Release of VEGF and TNFα was below quantifiable threshold in supernatants by ELISA, but we analyzed mRNA expression levels by qRT‐PCR. VEGF mRNA was increasingly expressed over time under mild hypoxia (5% O_2_), it was also upregulated after 4 and 24 hr exposure to severe hyperoxia (95% O_2_) and slightly upregulated under intermittent hyperoxia/hypoxia (Figure 1b). However, after 72 hr, constant and intermittent hyperoxia induced a return to control levels. TNFα expression slightly increased early under severe hyperoxia (Figure 1b). Most impressive was a strong upregulation under chronic (72 hr) exposure to hypoxia (3.79 ± 1.63 fold change vs. normoxia; *p* = .041) and intermittent hypoxia/hyperoxia (4.64 ± 1.99 fold change vs. normoxia; *p* = .034), while samples from constant hyperoxia stayed at control levels.

#### 3.1.2 Cell adhesion molecules

**FIGURE 2:**
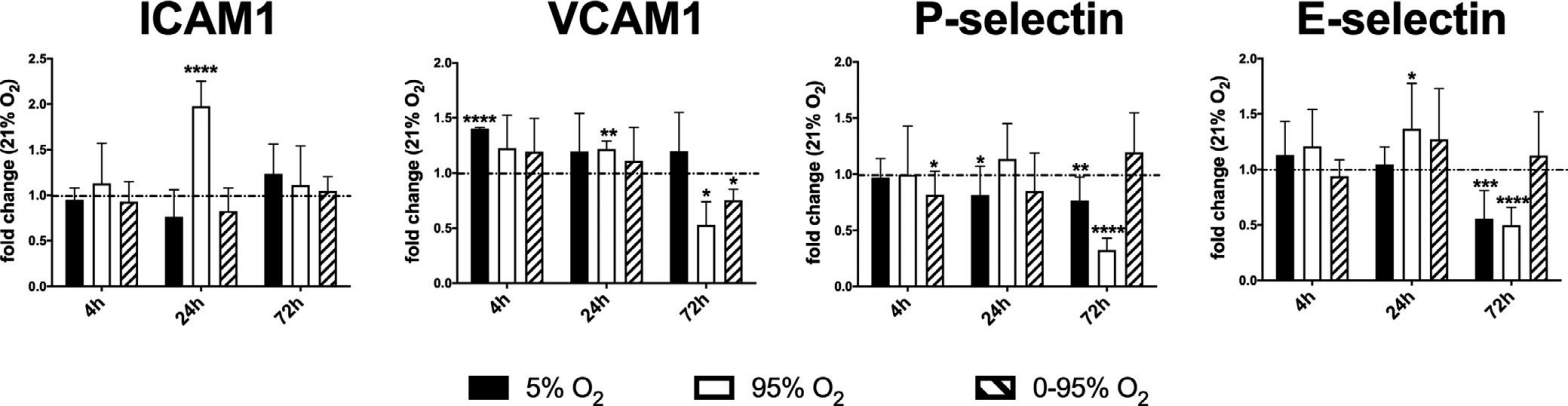
Expression of cell adhesion molecules (mRNA levels) in response to different oxygen conditions (fold change relative to control condition: 21% O_2_) as determined by qRT‐PCR from cell lysates using *actb* as house‐keeping gene and the ΔΔCt method. *p*‐values result from statistical testing with Student's *t*‐test: *p < .05; **p < .01; ***p < .001, ****p < .0001

ICAM1 (intercellular adhesion molecule) is a cell adhesion molecule that binds to leukocyte integrins (LFA‐1) in the course of diapedesis (Rahman & Fazal, 2009). Constant hyperoxia induced upregulation of ICAM1 mRNA after 24 hr to (1.98 ± 0.27) fold expression (*p* < .0001), as compared to normoxia, which returned to baseline at 72 hr (Figure 2). Intermittent hypoxia/hyperoxia did not induce changes in ICAM1 mRNA levels over all time points. VCAM1 (vascular cell adhesion molecule) is normally expressed after cytokine stimulation of the endothelium and binds to the β1 family of integrins (VLA‐4) on the surface of leukocytes (Lobb et al., 1996). VCAM1 mRNA expression was elevated under short‐term (4 hr) hypoxia (1.40 ± 0.01; *p* < .0001), 24 hr constant hyperoxia (1.22 ± 0.30; *p* = .007), but downregulated under chronic conditions (72 hr) of constant hyperoxia (0.53 ± 0.21; *p* = .017) or intermittent hypoxia/hyperoxia (0.53 ± 0.21; *p* = .0006 and 0.75 ± 0.10; *p* = .0003, respectively, as compared to normoxia, Figure 2). P‐ and E‐selectin are both members of the selectin family. They are calcium‐dependent lectins binding to glycoproteins like PSGL‐1 (including the sialyl Lewis X epitope) (Jin & Wang, 2020). While P‐selectin is usually stored in endothelial Weibel‐Palade bodies, E‐selectin expression is induced upon cytokine stimulation. In our setting P‐selectin was downregulated after 4 hr intermittent hypoxia/hyperoxia (0.81 ± 0.20; *p* = .046), and 72 hr of constant hypoxia (0.76 ± 0.21; *p* = .0189) and constant hyperoxia (0.32 ± 0.10; *p* < .0001, Figure 2). However, intermittent hypoxia/hyperoxia resulted in a tendency towards P‐selectin upregulation after 72 hr. E‐selectin expression was downregulated after 72 hr of constant hypoxia (0.56 ± 0.25; *p* = .0013) and constant hyperoxia (0.49 ± 0.16; *p* < .0001), and also showed a tendency of upregulation under chronic intermittent hypoxia/hyperoxia (Figure 2).

#### 3.1.3 Coagulation and fibrinolysis

**FIGURE 3:**
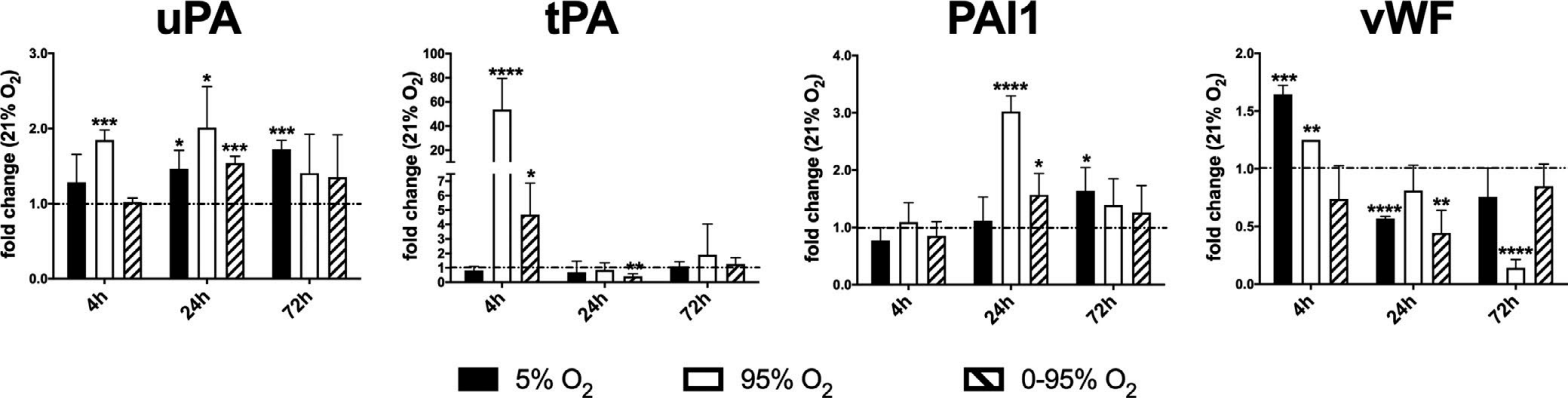
Expression of genes involved in coagulation and fibrinolysis (mRNA levels) in response to different oxygen conditions (fold change relative to control condition: 21% O_2_) as determined by qRT‐PCR from cell lysates using *actb* as house‐keeping gene and the ΔΔCt method. *p*‐values result from statistical testing with Student's *t*‐test: *p < .05; **p < .01; ***p < .001, ****p < .0001

Plaminogen activators urokinase (uPA) and tissue‐type (tPA) convert plasminogen to fibrinolytically active plasmin. Plasminogen activator inhibitor (PAI1) is counteracting this process by inhibiting uPA and tPA (Tucker & Idell, 2013). In this study, uPA mRNA expression was upregulated already in short‐term constant hyperoxia (4 hr: 1.85 ± 0.14; *p* < .0001; 24 hr: 2.01 ± 0.55; *p* = .033) (Figure 3). At 24 hr, constant hypoxia and intermittent hypoxia/hyperoxia resulted in increased uPA expression (1.46 ± 0.25; *p* = .0016 and 1.54 ± 0.09; *p* = .0018, respectively, as compared to normoxia). At 72 hr uPA was only increased under constant hypoxia (1.72 ± 0.11; *p* < .0001). tPA was rapidly upregulated by constant hyperoxia and intermittent hypoxia/hyperoxia (4 hr: 52.02 ± 43.46; *p* = .0125 and 4.09 ± 2.16; *p* = .0028, respectively, Figure 3) and returned to lower than basal levels after that (intermittent hypoxia/hyperoxia: 0.43 ± 0.14; *p* < .0001). PAI1 was upregulated after 24 hr of constant hyperoxia and intermittent hypoxia/hyperoxia (3.02 ± 0.27; *p* < .0001 and 1.57 ± 0.37; *p* = .0043, respectively, Figure 3). At 72 hr PAI1 mRNA expression was only significantly increased in cells kept under hypoxic conditions (1.64 ± 0.41; *p* = .005). Von Willebrand factor (vWF) plays an important role in blood coagulation as a binding protein for Factor VIII and in thrombocyte adhesion at wounds. vWF is usually stored in endothelial Weibel Palade bodies that are however absent in pulmonary capillaries ( Ochoa, Wu, & Stevens, 2010). vWF was rapidly upregulated under constant hypoxia and hyperoxia (4 hr: 1.64 ± 0.07; *p* < .0001 and 1.25 ± 0.01; *p* < .0001, respectively, as compared to normoxia, Figure 3), but later downregulated under hypoxia (24 hr: 0.57 ± 0.02; *p* < .0001), intermittent hypoxia/hyperoxia (24 hr: 0.44 ± 0.19; *p* = .0001) and constant hyperoxia (72 hr: 0.14 ± 0.07; *p* < .0001).

#### 3.1.4 Vasoactive mediators

**FIGURE 4:**
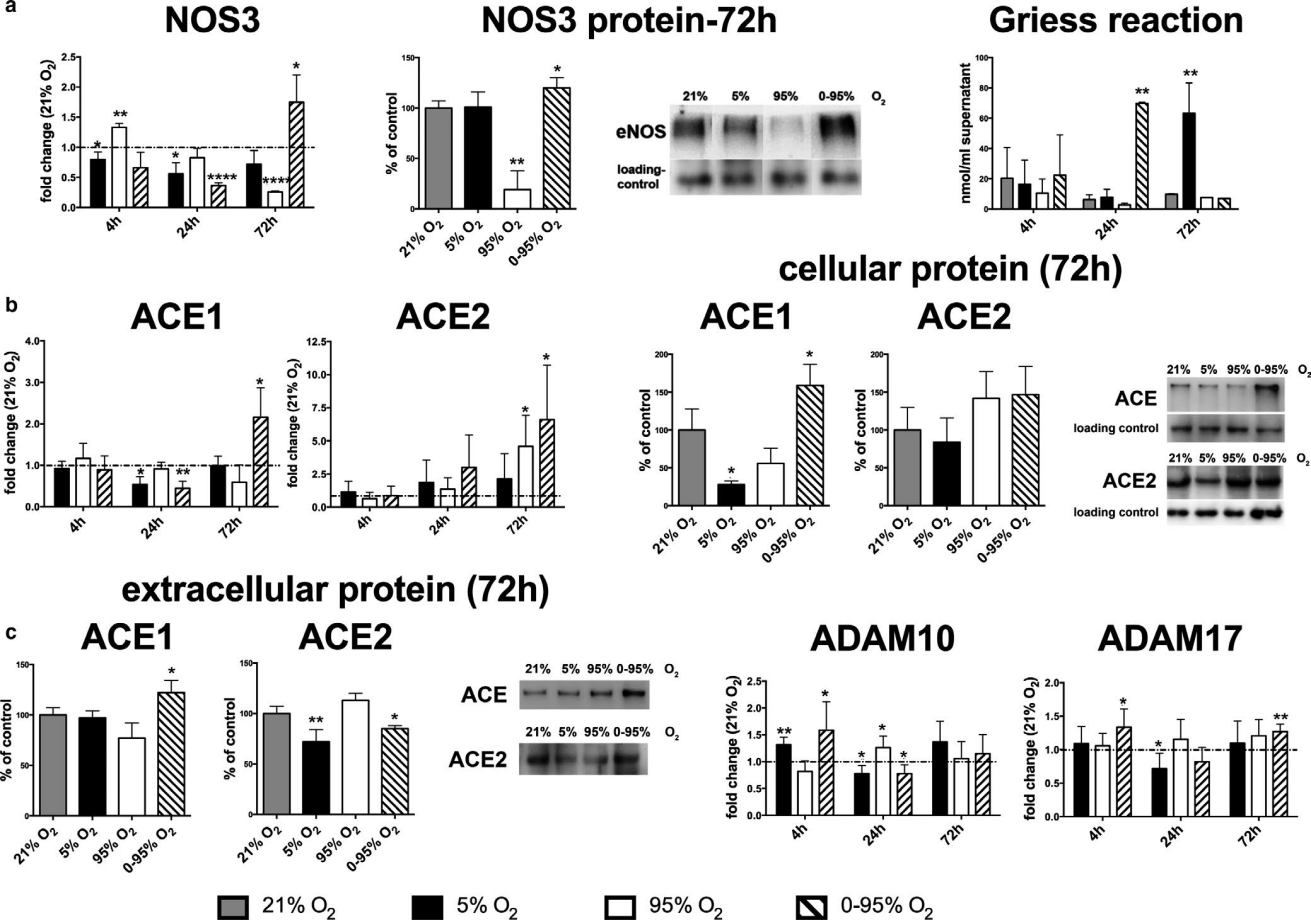
Expression of vasoactive proteins (mRNA and protein levels) in response to different oxygen conditions (mRNA: fold change relative to control condition: 21% O_2;_ protein: % of control condition: 21% O_2_) as determined by qRT‐PCR using *actb* as house‐keeping gene and the ΔΔCt method and western blotting from cell lysates. (a) NOS3 mRNA expression, protein expression at 72 hr and Griess reaction (analyzed levels of NO_2_^−^ and NO_3_^−^ in the supernatants resulting from NOS activity). (b) ACE1, ACE2 mRNA expression and ACE1, ACE2 intracellular protein expression at 72 hr. (c) ACE1 and ACE2 protein levels in the cell culture supernatant as determined by Western blotting and mRNA expression of sheddases ADAM10 and ADAM17. *p*‐values result from statistical testing with Student's *t*‐test (mRNA), one‐way ANOVA (protein) and two‐way ANOVA (Griess) with Tukey's multiple comparison test. *p < .05; **p < .01; ***p < .001, ****p < .0001

NOS3 (eNOS) is the constitutively expressed endothelial isoform of nitric oxide synthase (Shu & Keller TCt et al.., 2015). NOS3 mRNA was changed at 4 hr of hypoxia (0.79 ± 0.12; *p* = .0038) and constant hyperoxia (1.33 ± 0.07; *p* < .0001), and downregulated at 24 hr in hypoxia (0.56 ± 0.18; *p* = .0004) and intermittent hypoxia/hyperoxia (0.36 ± 0.05; *p* < .0001, Figure 4a). At 72 hr the enzyme's mRNA expression was severely downregulated under constant hyperoxia (0.26 ± 0.01; *p* < .0001) and upregulated under intermittent hypoxia/hyperoxia (1.75 ± 0.45; *p* = .0034). After 72 hr of constant hyperoxia, protein levels of NOS3 were reduced to (19 ± 19%; *p* = .0023) of normoxic levels (Figure 4a). After 72 hr of hypoxic/hyperoxic oscillations NOS3 protein levels were upregulated to (120 ± 10%; *p* = .047) versus normoxia (Figure 4a).

Nitric oxide synthase produces NO from the amino acid L‐arginine. NO however has a short physiological half‐life and is primarily converted to other compounds by reactive oxygen species. Major break‐down products are nitrite and nitrate which can be determined by the Griess reaction (Bevers, 2006). In our experimental set‐up the color intensity of the formation of the red azo‐dye was strongest after 24 hr of intermittent hypoxia/hyperoxia (11.12 ± 0.09 fold upregulation, as compared to normoxia) and 72 hr of constant hypoxia (6.45 ± 2.04 fold upregulation, as compared to normoxia) (Figure 4a).

Angiotensin converting enzyme (ACE) 1 is a main component of the renin‐angiotensin system (RAS) and has a central function in the control of fluid balance and blood pressure by converting the hormone angiotensin (Ang) I to Ang II. The largest amounts of this enzyme are located in the pulmonary capillaries, although its role in the lung is not fully understood yet (Tan, Liao, Zhou, Mei, & Wong, 2018). In our setting, the mRNA expression of ACE1 remained unaltered or low under all oxygen conditions until 24 hr (hypoxia: 0.54 ± 0.18; *p* = .0003; intermittent hypoxia/hyperoxia: 0.45 ± 0.17; *p* = .0034, as compared to normoxia, Figure 4b). At 72 hr only intermittent hypoxia/hyperoxia lead to a strong upregulation of ACE1 (2.16 ± 0.70; *p* = .003). ACE2 is a newly discovered enzyme counteracting ACE1 by cleavage of the ACE1 product Ang II to Ang 1–7 (Jia, 2016). While Ang II may promote lung fibrosis and apoptosis, Ang 1–7 has anti‐fibrotic, anti‐inflammatory and anti‐apoptotic properties. Significant changes in ACE2 mRNA expression were detected after 72 hr of severe hyperoxia (4.58 ± 2.34 fold change; *p* = .0091) and intermittent hypoxia/hyperoxia (6.61 ± 4.11 fold change; *p* = .0145) (Figure 4b). In both cases, changes in ACE1/2 mRNA levels were also partly reflected in cellular protein levels (Figure 4b). At 72 hr, ACE1 protein levels in cellular extracts had decreased to (28 ± 5%) of protein levels at 21% O_2_ under hypoxia (*p* = .012) and (56 ± 20%) under constant hyperoxia (*p* = .09), but increased to (159 ± 28%) under intermittent hypoxia/hyperoxia (*p* = .06). Similarly, cellular ACE2 protein levels had a tendency to increase after 72 hr of constant hyperoxia (142 ± 35%; *p* > .05; as compared to 21% O_2_) and intermittent hypoxia/hyperoxia (147 ± 37%, *p* > .05; as compared to 21% O_2_).

It needs to be considered, that ACE1/2 protein activity is further regulated by proteolytic shedding from the cell surface. We therefore quantified ACE1 and ACE2 protein levels in cell culture supernatants after 72 hr of gas exposure and found higher levels of ACE1 under intermittent hypoxia/hyperoxia (122 ± 12%; *p* = .05; as compared to 21% O_2_) and reduced levels of ACE2 after 72 hr of hypoxia (72 ± 12%; *p* = .025; as compared to 21% O_2_) and intermittent hypoxia (85 ± 3%; *p* = .027; as compared to 21% O_2_) (Figure 4c). A function as shedding proteases is attributed among others to the ADAM family of transmembrane metalloproteases. The best‐described representatives are ADAM10 and ADAM17 which have been especially characterized with regard to ACE2 shedding (Grobe et al., 2015). In our setting mRNA levels of both enzymes were increased early after 4 hr of intermittent hypoxia/hyperoxia (ADAM10: 1.58 ± 0.54; *p* = .049; as compared to normoxia. ADAM17: 1.34 ± 0.27; *p* = .043; as compared to normoxia). ADAM17 was also elevated at 72 hr in this group (1.27 ± 0.11; *p* = .043, Figure 4c).

#### 3.1.5 Other factors

**FIGURE 5:**
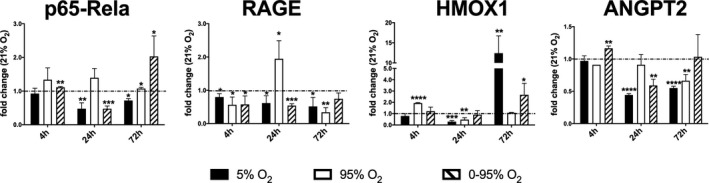
Expression of other marker genes (mRNA levels) in response to different oxygen conditions (fold change relative to control condition: 21% O_2_) as determined by qRT‐PCR from cell lysates using *actb* as house‐keeping gene and the ΔΔCt method. *p*‐values result from statistical testing with Student's *t*‐test: *p < .05; **p < .01; ***p < .001, ****p < .0001

A main component of the transcription factor NFKB is p65 (Rela) which complexes with p50 as the most prevalent dimeric form of the transcription factor. Although activation of the pathway is mainly regulated by various phosphorylation events, expression levels of NFKB components have been shown to have an impact on its signaling (Wang & Cassidy, 2003). P65 is downregulated after 24 hr of hypoxia (0.47 ± 0.18; *p* = .068) and intermittent hypoxia/hyperoxia (0.47 ± 0.08; *p* = .0004), but slightly upregulated under constant hyperoxia (1.40 ± 0.27; *p* = .07). However, after 72 hr of hypoxic/hyperoxic oscillations mRNA levels were upregulated to (2.03 ± 0.61 fold; *p* = .048), while under other conditions mRNA levels returned to baseline (Figure 5).

The receptor for advanced glycation end products (RAGE) is a multifunctional protein, whereby its role in inflammation is best studied. RAGE is highly expressed in lungs in a developmental stage dependent manner and has been shown to play a role in many lung diseases (Oczypok, Perkins, & Oury, 2017). Similar to other plasma membrane proteins it can be shedded by proteases (ADAM10, matrix metalloprotease 9) to form the soluble decoy receptor sRAGE. RAGE knock‐out mice have been shown to be protected from HALI (Reynolds et al., 2010). After 4 hr of constant hyperoxia and intermittent hypoxia/hyperoxia RAGE mRNA expression was downregulated, as compared to normoxia (hyperoxia: 0.56 ± 0.23; *p* = .038; O_2_ oscillations: 0.57 ± 0.25; *p* = .044). After 24 hr of constant hyperoxia RAGE was strongly upregulated (1.95 ± 0.54; *p* = .039) and at 72 hr again downregulated (0.34 ± 0.13; *p* = .001). Hypoxic/hyperoxic oscillations did not stimulate RAGE mRNA expression over the whole observed time period (Figure 5).

Heme oxygenase (HMOX) is an enzyme catalyzing the rate‐limiting step in the break‐down of heme, thereby generating carbon monoxide (CO) and biliverdin‐IXα. The induction of the inducible isoform HMOX1 is mostly regarded a protective measure to reduce or modulate inflammatory and apoptotic processes and has also been demonstrated to have beneficial effects in lung diseases (Constantin, Choi, Cloonan, & Ryter, 2012). In our microvascular endothelial cells HMOX1 mRNA expression was rapidly upregulated under constant hyperoxia at 4 hr (1.93 ± 0.05; *p* < .0001, vs. 21% O_2_) and was later reduced at 24 hr (0.48 ± 0.17; *p* = .058). At 72 hr HMOX1 was massively upregulated under constant hypoxia (12.43 ± 4.28; *p* = .009), but also under intermittent hypoxia/hyperoxia (2.67 ± 1.02; *p* = .046, Figure 5).

Angiopoietins regulate vascular homeostasis, whereby angiopoietin (ANGPT)‐1 stabilizes the endothelium and ANGPT‐2 sometimes merely facilitates vascular permeability, inflammation and other pathological developments (Augustin, Koh, Thurston, & Alitalo, 2009). ANGPT2 was only altered at 24 hr under hypoxic and intermittent hypoxic/hyperoxic oxygen conditions (hypoxia: 0.44 ± 0.02; *p* < .0001; oscillations: 0.59 ± 0.09; *p* = .0018; as compared to 21% O_2_) (Figure 5).

#### 3.1.6 Redox systems and oxidative stress

**FIGURE 6:**
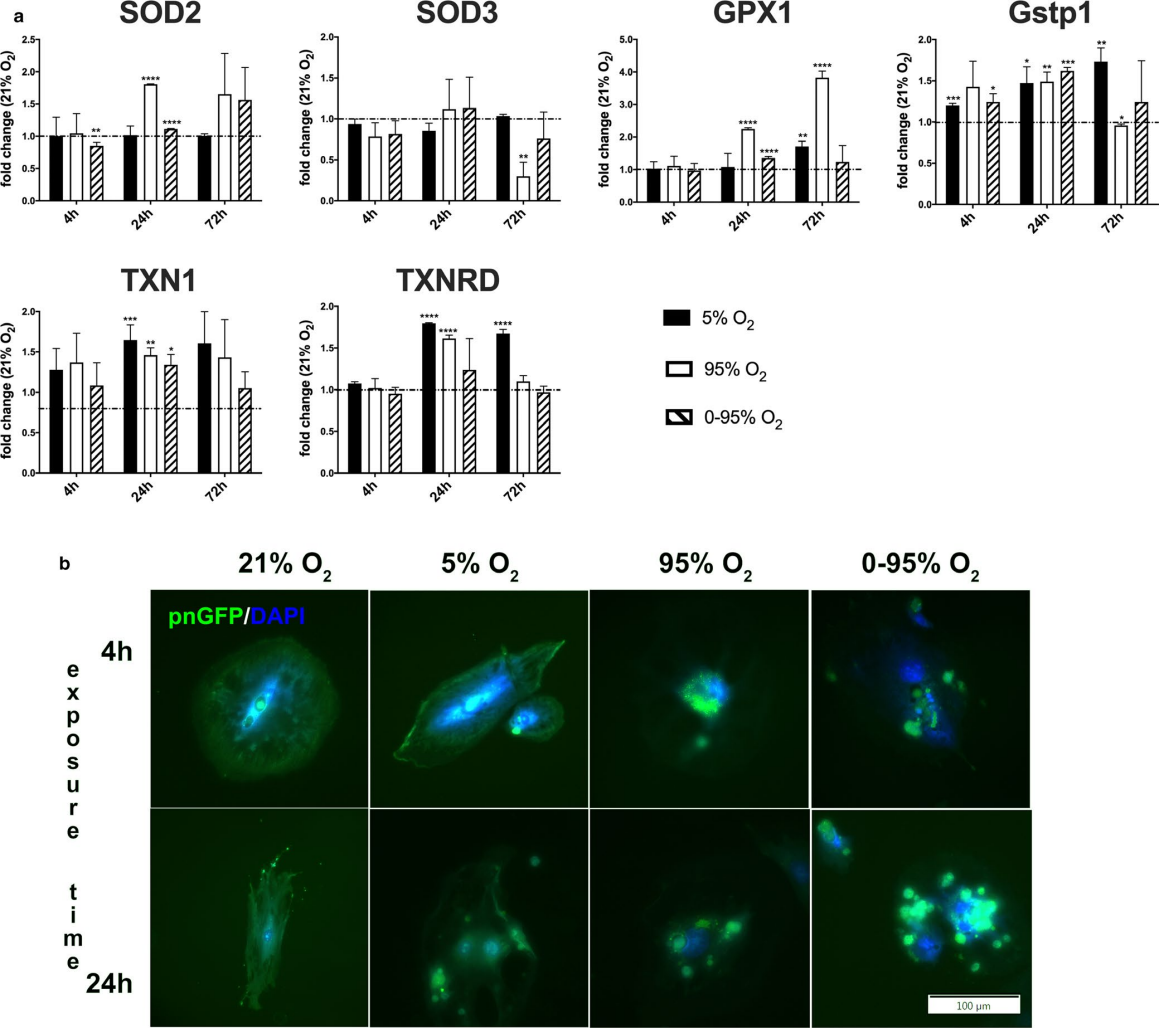
Effects on different oxygen conditions on genes involved in regulating oxidative stress. (a) Expression levels of genes buffering oxygen radicals (fold change relative to control condition: 21% O_2_) as determined by qRT‐PCR from cell lysates using *actb* as house‐keeping gene and the ΔΔCt method. *p*‐values result from statistical testing with Student's *t*‐test: *p < .05; **p < .01; ***p < .001, ****p < .0001. (b) Imaging of MLECs expressing pnGFP exposed to different oxygen conditions for 4 and 24 hr. pnGFP only exhibits green fluorescence upon reaction with peroxynitrite. DAPI (blue) was used to visualize cell nuclei. Scale bar: 100 μm

An imbalance of the oxygen homeostasis is frequently reflected in a change of expression of proteins involved in ROS detoxification. We have selected some key enzymes involved in the dissipation of superoxide, H_2_O_2_, and other radicals, checking their mRNA expression levels under different oxygen conditions over time (Figure 6a). The mitochondrial superoxide dismutase SOD2 was slightly downregulated after 4 hr of intermittent hypoxia/hyperoxia (0.85 ± 0.05; *p* = .0002; vs. 21% O_2_), and was upregulated after 24 and 72 hr of constant hyperoxia (24 hr: 1.80 ± 0.006; *p* < .0001; 72 hr: 1.66 ± 0.62; *p* = .021) and intermittent hypoxia/hyperoxia (24 hr: 1.12 ± 0.007; *p* < .0001; 72 hr: 1.56 ± 0.50; *p* = .015). Extracellular superoxide dismutase (SOD3) was slightly downregulated after 4 hr of intermittent hypoxia/hyperoxia (0.76 ± 0.16; *p* = .011), 24 hr of constant hypoxia (0.85 ± 0.09; *p* = .003) and significantly downregulated after 72 hr of constant hyperoxia (0.30 ± 0.17; *p* < .0001).

Thioredoxin (TXN1) had a tendency for early upregulation at 4 hr which became statistically significant after 24 hr of hypoxia (1.65 ± 0.19; *p* < .0001), 24 hr of hyperoxia (1.46 ± 0.09; *p* < .0001), 24 hr of intermittent hypoxia/hyperoxia (1.34 ± 0.13; *p* = .0002), and 72 hr of hypoxia (1.61 ± 0.39; *p* = .046) as well as 72 hr of hyperoxia (1.43 ± 0.46; *p* = .043), while after 72 hr of intermittent hypoxia/hyperoxia levels returned to baseline. Thioredoxin reductase (TXNRD) is essential for the regeneration of TXN and the reduction of disulfides (Balsera & Buchanan, 2019). At 24 hr TXNRD3 was upregulated under hypoxia (1.61 ± 0.04; *p* < .0001) and hyperoxia (1.24 ± 0.37; *p* < .0001) and stayed upregulated until 72 hr under hypoxia (1.67 ± 0.05; *p* < .05) and only slightly yet still significantly under hyperoxia (1.10 ± 0.007; *p* = .049). Glutathione peroxidase (GPX1) is an important hydrogen peroxide detoxicant using glutathione as reductant (Brigelius‐Flohe & Maiorino, 1830). GPX1 is upregulated after 24 hr of constant hyperoxia (2.25 ± 0.03; *p* < .0001), 24 hr of intermittent hypoxia/hyperoxia (1.36 ± 0.03; *p* < .0001) and 72 hr of hypoxia (1.71 ± 0.16; *p* < .0001) and hyperoxia (3.83 ± 0.19; *p* < .0001). Glutathione S‐transferase P (Gstp1) is involved in detoxification by coupling reduced glutathione to other compounds (Vasieva, 2011). Expression of Gstp1 was increased early under all conditions (4 hr hypoxia: 1.20 ± 0.02; *p* < .0001, 4 hr hyperoxia: 1.43 ± 0.31; *p* = .009, 4 hr intermittent hypoxia/hyperoxia: 1.24 ± 0.10; *p* = .0004; as compared to normoxia), and further increased after 24 hr (hypoxia: 1.47 ± 0.19; *p* = .0004, hyperoxia: 1.49 ± 0.11; *p* < .0001, intermittent hypoxia/hyperoxia: 1.62 ± 0.04; *p* < .0001, as compared to normoxia). After 72 hr only hypoxia further increased the expression of Gstp1 (1.73 ± 0.16; *p* < .0001, vs. normoxia), while other conditions resulted in a decrease to almost baseline levels.

Cells have a large and cell type‐typical repertoire of antioxidative compounds including small molecules and also proteins, that dissipate and detoxify oxidative stress, which is mostly mediated by reactive oxygen species (ROS). These radicals display high reactivity toward cellular components, thereby altering protein and lipid properties (structure and function). Endothelial cells produce large amounts of nitric oxide, which diffuses readily through the membranes and together with superoxide transforms into peroxynitrite, which can have detrimental impact on many cellular targets. We used a peroxynitrite‐sensitive GFP variant to visualize the amount and subcellular location of its generation in response to the different oxygen conditions (Figure 6b). Constant and even more intermittent hyperoxia displayed high amounts of peroxynitrite generation (most probably near mitochondria), which started as early as after 4 hr exposure and was strongest after 24 hr exposure.

## 4 DISCUSSION

Ventilation with high FiO_2_ levels is frequently necessary in intensive care to achieve sufficient oxygenation in hypoxemic conditions. A much too liberal oxygen application has raised considerable criticism in the recent years, as the detrimental effects of oxygen toxicity have been previously underestimated. Severe oxygen‐induced injury is subsumed under the term HALI and can complicate the condition of critically ill patients, who receive supplemental oxygen therapy. The lung is at the forefront of encountering high oxygen concentrations which damage the alveolar epithelium and also swiftly reach the vasculature. Due to recruitment and de‐recruitment of atelectasis (which might be further aggravated due to absorption atelectasis) high oxygen concentrations might be “intermittent,” resulting in oxygen oscillations that are transmitted via the bloodstream to remote tissues.

The pulmonary endothelium lines the macro‐ and microvascular structures in the lung and regulates vascular tone, coagulation, leukocyte diapedesis, and further barrier functions for fluid and proteins. Many of these functions are disrupted in lung diseases such as ARDS (Millar, Summers, Griffiths, Toshner, & Proudfoot, 2016). Also under physiological conditions the pulmonary vasculature experiences the broadest range of oxygen concentrations, as it transports deoxygenated and fully re‐oxygenated blood. In this study, we were particularly interested in the response of the lung endothelium to oxygen conditions oscillating between hypoxia and hyperoxia, as the activation state or mediators of injury of the endothelium can have important downstream functional implications that may be clinically relevant in ventilated patients. Our experiments show that the changes in molecular patterns induced by intermittent hypoxia/hyperoxia, constant hypoxia and constant hyperoxia involve important aspects of endothelial cell function, such as cytokine release, expression of cell adhesion molecules, regulation of coagulation, fibrinolysis and vascular tone as well as redox systems.

Transmission of breathing gas via the alveoli to the lung microvasculature is rapid and only weakly muted. In our study, we simulated this situation by using cell culture plates with gas‐permeable membranes as growth support. The gas approaches the cells from underneath and passes a membrane to reach the cells without the need to first dissolve in a growth medium.

Effects of constant hyperoxia have been investigated in many studies with different focus (Dias‐Freitas, Metelo‐Coimbra, & Roncon‐Albuquerque, 2016). Similarly, also constant and intermittent hypoxia has attracted broad interest, as those are hallmarks in several disease states (Semenza, 2006). These conditions all induce oxidative stress, albeit via their specific molecular pathways. Only very few studies provide insight into molecular mechanisms, that play a role in conditions of combined intermittent hypoxia/hyperoxia. In our study, we directly compared responses of lung endothelial cells to typical hypoxic, normoxic, hyperoxic, and intermittent hypoxic/hyperoxic conditions. We used expression analyses to monitor cell responses in the short term (4 hr), intermediate (24 hr) and long‐term (72 hr). Beyond 72 hr oxygen toxicity in the 95% constant O_2_ group already leads to significant cell death as revealed by the increase of LDH release.

*Constant hyperoxia* is known to induce inflammation (Dias‐Freitas et al., 2016). In our model constant severe hyperoxia increased the release of pro‐inflammatory cytokines like KC/CXCL1 and MIP2/CXCL2 after 72 hr, and induced enhanced expression of ICAM1, VCAM1, E‐selectin, and RAGE at 24 hr. NFKB p65 was slightly upregulated from 4 hr onwards, which might explain the increase in pro‐inflammatory cytokines. Dysregulation of coagulation and fibrinolysis was observed with an early upregulation of tPA, uPA, and vWF at 4 hr, upregulation of uPA and PAI1 at 24 hr and massive downregulation of vWF at 72 hr. NOS3 mRNA was rapidly upregulated under constant hyperoxia (4 hr) and downregulated at later time points. NOS enzymatic activity was already reduced at the earliest time point, as revealed by the Griess reaction, possibly due to oxidative uncoupling of the enzyme. This result allows a more differentiated conclusion compared to the study of Attaye et al. (2017). In our study NOS3 expression persisted until 24 hr, after that mRNA and protein levels declined until 72 hr. However, enzyme activity was already compromised at the earliest time point (4 hr), which might at least partly explain hyperoxic vasoconstriction that is observed in many vascular beds. With regard to the RAS enzymes, we only observed an upregulation of ACE2 at 72 hr under hyperoxia. Furthermore, hyperoxia leads to the induction of several antioxidative enzymes, including SOD2, TXN1, TXNRD3, Gstp1, and GPX1 at 24 hr. GPX1 is especially interesting, as its expression gradually increased over the three time points, while the expressions of the other enzymes decreased again at 72 hr.

*Constant hypoxia* induced a typical increase in VEGF mRNA expression, but also expression of pro‐inflammatory markers like VCAM1 at 4 hr, and TNFα and MIP2/CXCL2 at 72 hr. Hypoxia induced increased expression of uPA from 24 hr onwards, PAI1 at 72 hr and a decrease of vWF from 24 hr onwards. NOS3 was downregulated from 4 hr onwards, as were ACE1, NFKB p65, HMOX1, and ANGPT2 at 24 hr. In response to hypoxia‐induced oxidative stress TXN1, TXNRD3, and Gstp1 were upregulated from 24 hr onwards and GPX1 was upregulated at 72 hr.

*Intermittent hypoxia/hyperoxia* reveals a characteristic gene expression signature that is different from hypoxic or hyperoxic conditions. Seventy‐two hours of exposure did not compromise viability of endothelial cells as implicated by comparable LDH release to normoxia. There were also less indicators of a pro‐inflammatory state as under constant hyperoxic conditions as deduced from the release of pro‐inflammatory cytokines or the expression of cell adhesion molecules that would facilitate adherence or diapedesis of pro‐inflammatory blood cells. Only a marked increase in TNFα expression at 72 hr was prominent. There is also evidence of changes in the coagulative state of the endothelium, as uPA and PAI1 were upregulated, and vWF was downregulated at 24 hr. Most interesting were changes in the expression (and activity) of vasoactive factors, including NOS3 and the RAS enzymes. NOS3 activity seems to be upregulated until 24 hr, as NO metabolites quantified by the Griess reagent from the supernatant increased until this time point, while mRNA levels declined (probably as a compensatory mechanism). At 72 hr, however, enzyme activity seems to have vanished and mRNA levels increased significantly.

Important biologically active angiotensin metabolites are generated by the enzymes ACE1 and ACE2, which have counteracting functions. ACE1 mediates formation of Ang II, a potent vasoconstrictor acting through Ang II receptors type 1 and 2 that has also been shown to promote fibrosis and apoptosis in the lung, whereas ACE2 cleaves Ang II to Ang 1–7 that is described as a lung‐protective molecule acting through the Mas receptor (Santos et al., 2018; Uhal, Li, Xue, Gao, & Abdul‐Hafez, 2011). While ACE1 was slightly downregulated at 24 hr, it was strongly upregulated at the later time point (72 hr). In parallel, ACE2 was also upregulated. mRNA regulation is also reflected in amounts of cellular protein, but activity of these enzymes is further post‐translationally regulated by shedding of the extracellular peptides from the cell surface. When analyzing released proteins in the supernatant, we found increased levels of ACE1, but decreased levels of ACE2 at 72 hr. Candidate sheddases ADAM10 and ADAM17 were upregulated early (4 hr) under intermittent hypoxia/hyperoxia unlike other oxygen conditions. In the light of the current pandemic of COVID 19, it is of significant interest, that oxygen oscillations, as might be occurring during mechanical ventilation with supraphysiological oxygen concentrations activate the RAS (i.e., especially upregulate ACE). It can be assumed, that this might have even more impact, when ACE2 is hijacked by the virus, which could disbalance the system. In general, the differential expression and activity of NOS3 and the RAS enzymes during different oxygen conditions gives a complex picture and demand animal experiments to analyze their impact on vascular tone.

NFKBp65 showed triphasic dynamics: It was slightly upregulated at 4 hr, significantly down‐regulated at 24 hr and strongly upregulated at 72 hr. RAGE mRNA levels remained below normoxic levels over all time points. HMOX1 was upregulated at 72 hr. The endothelial injury marker ANGPT2, that regulates vascular permeability (and in plasma is used as a prognostic mortality marker in ARDS (Calfee et al., 2012)) was upregulated at 4 hr and downregulated at 24 hr.

Oxygen oscillations in different oxygen ranges have been shown to generate significant ROS. As a gene expression response of the antioxidative systems, apart from upregulation of Gstp1 and TXN1, all other responses are dampened compared to other oxygen conditions. Of special interest is the fact that oxygen oscillations obviously result in production of a huge amount of peroxynitrite in these endothelial cells. Downstream reactions of this radical are pleiotropic and include various modifications of aminoacids resulting in changes of enzymatic activities, structure and protein aggregation, which can underlie a multitude of disease states (Moreno & Pryor, 1992; Trujillo & B. et al., 2010). The fast reaction between superoxide and nitric oxide outcompetes detoxification by superoxide dismutases. These enzymes (SOD1 and SOD2) are further deactivated by this radical, as well as tetrahydrobiopterin, the cofactor of nitric oxide synthases, thereby causing NOS uncoupling leading to increased superoxide production. Peroxynitrite generation mostly takes place at the sites of superoxide generation, which‐in contrast to nitric oxide‐ cannot diffuse as readily. With regard to the lung, of special importance is the fact, that peroxynitrite inactivates alpha 1‐antitrypsin by oxidizing a crucial methionine, that can lead to emphysema (Moreno & Pryor, 1992). It has been further shown, that peroxynitrite also affects kinases and phosphatases, that regulate endothelial barrier function, such as Akt, PP2A, PTP1B, and PTEN (Delgado‐Esteban, Martin‐Zanca, Andres‐Martin, Almeida, & Bolanos, 2007; Wu & Wilson, 2009). A further important impact on signaling is nitration of the NF‐KB inhibitor IKBα, leading to disruption of the inhibitor binding and an increase in the activity of this transcription factor (Yakovlev et al., 2007). This is further interesting in the light of mRNA upregulation of the NF‐KB subunit p50 under long‐term exposure to hypoxic/hyperoxic O_2_ oscillations. NF‐KB activation increases inflammation, but also contributes to cell survival as an anti‐apoptotic factor. This might explain, why the viability of the lung endothelial cells does not suffer much from oxygen oscillations. Nitrated proteins frequently also form a new epitope for immunoglobulins, which have for instance been found to be elevated in patients with acute lung injury. As peroxynitrite leaves typical marks on several proteins, it can be suggested, that it could be worthwhile to investigate into their potential use as biomarkers for the severity of peroxynitrite formation.

### 4.1 Limitations of the study

Generally, endothelial cells have tissue‐specific properties. Even within the lung, the heterogeneous population of pulmonary endothelial cells poses a challenge to vascular biologists (Stevens et al., 2008). Our study was designed as an in vitro cell culture study, where endothelial cells were isolated from whole mouse lungs in order to monitor cell responses from a cell population of endothelial origin. We acknowledge that these cells are still a mixed pool of endothelial cells from larger and smaller vessels. However, the majority of endothelial cells are located in the microvasculature of the lung and those cells also have an exceptionally high proliferative potential (as an intrinsic property and also due to an enrichment in progenitor cells) (Clark et al., 2008). Therefore, these cells can be anticipated to predominate in cell culture.

Despite the advantage of an in vitro system, where isolated cell responses can be observed under controlled conditions and molecular mechanisms can be elucidated more easily due to the reduced complexity, such systems ignore influencing factors from other cell types, and the fact, that the observed cell type might undergo culture‐specific transformations. A further limitation of this study is the gas transition time through the membrane that limits the achievable oscillation frequencies, which would need to be much higher in order to realistically simulate ventilation. In order to realistically model mechanical ventilation with different oxygen concentrations and patterns, it is also necessary to consider, that the mechanical force during this intervention imposes shear stress onto tissues, which can be aggravated by reorganization of the cytoskeleton due to oxidative stress as occurring under hyperoxia. According experimental systems are available, where cells are cultured in special chambers, where they can be stretched and simultaneously exposed to different gases at the same time (Tretter et al., 2020). It has been shown, that hyperoxia can induce polymerization of actin or, as in endothelial cells, an increase in actin stress fibers, thereby affecting cellular stiffness ((Roan et al., 2012) and references therein). In order to reduce cell injury, the basement membrane should deform in a similar way during stretch in order to avoid damage of focal adhesions and intercellular junctions, implicating that substrate stiffness is another influencing factor. Further experimental approaches should aim to take into account these variables when simulating oxygen exposure in the context of mechanical stretch. In this study, however, we aimed to measure cell responses, which are solely attributed to the influence of oxygen conditions. Significant differences of the gene expression signature in response to different oxygen conditions and patterns indicate the validity of the model and the feasibility of the approach to address the research question.

## 5 CONCLUSIONS

Pulmonary endothelial cells show specific molecular responses when exposed to oxygen oscillations in the hypoxic/hyperoxic range, as they might occur in the ventilated diseased lung under supplemental oxygen therapy that have the potential to induce or contribute to pathological developments. The effects on gene expression were time‐dependent and included targets involved in inflammation and regulation of coagulation, fibrinolysis, vascular tone, and redox systems, that is, principal functions of endothelial cells. To further analyze the impact of these molecular responses on the physiology of a living organism according experiments in healthy animals are required in order to exclude confounding co‐morbidities that occur in ventilated patients. Also, a detailed understanding of molecular events will help to establish tailored pharmacological therapies to counteract oxygen toxicity in the future.

## DISCLOSURES

The authors declare no conflict of interest related to the content of this paper. Outside the submitted work KK and RU report personal fees (travel grants) from Biotest and grants from Apeptico. RU holds equity in CCORE Technology, a medical device company that develops minimally invasive blood purification devices.

## ACKNOWLEDGMENTS

We thank the Corefacility Imaging, especially Marion Gröger and Christoph Friedl for the support during this study.

## AUTHORS’ CONTRIBUTIONS

PW, KUK, and VT designed the study. KUK, KM, and VT designed parts of the experimental set‐up. PW, JMD, WS, AT, and VT performed experiments and analyzed data. KK, RU, and KM had an advisory role. KK and VT wrote the manuscript.

## Supporting information



Fig S1

Fig S2

Fig S3
