# Supplementary figures and images for: Oxygen conditions oscillating between hypoxia and hyperoxia induce different effects in the pulmonary endothelium compared to constant oxygen conditions
Source: Physiol Rep. 2021 Feb 10;9(3):e14590. doi: 10.14814/phy2.14590 (PMC7873712; doi:10.14814/phy2.14590)

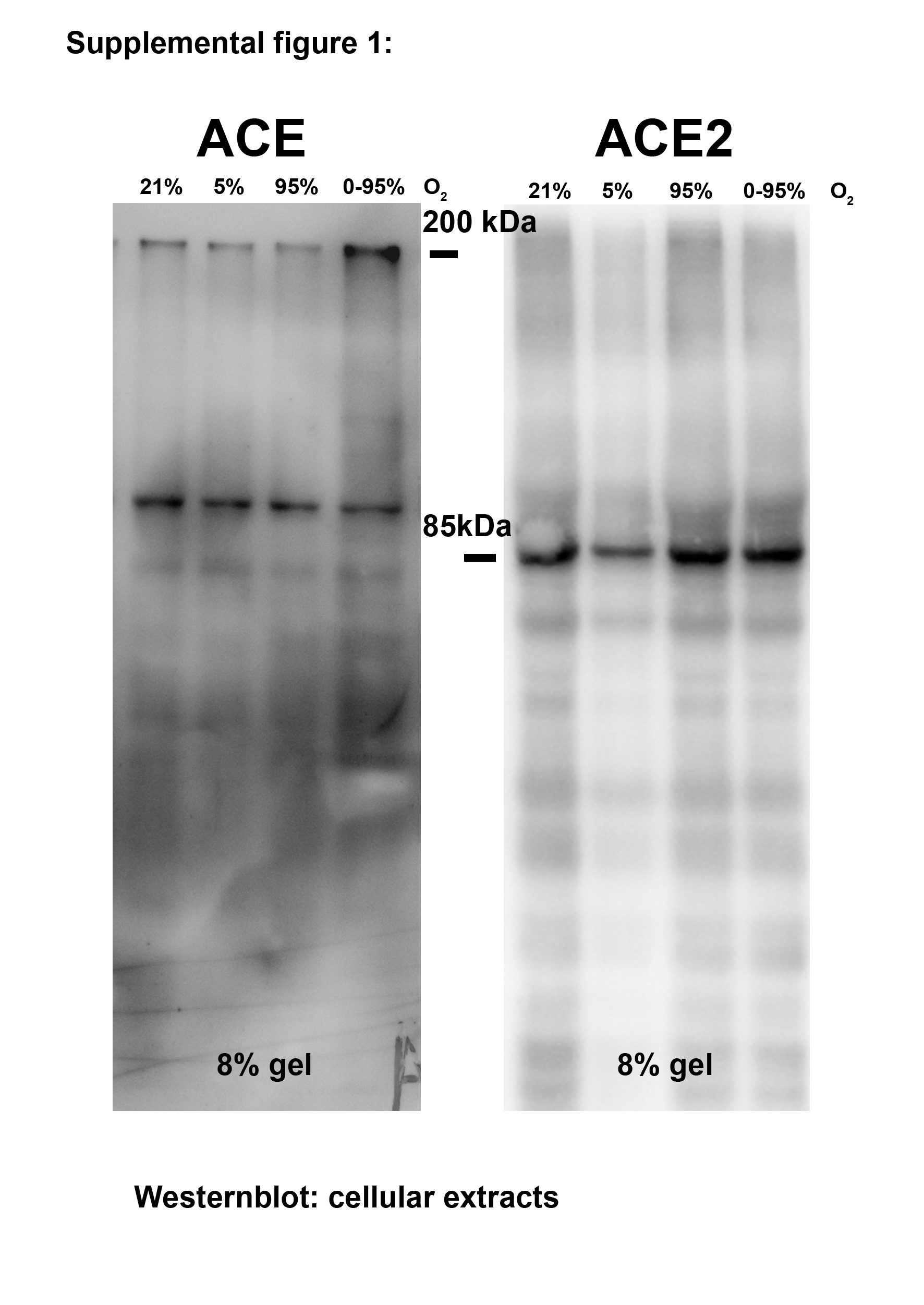

Supplement: Supplementary file 1 — Fig S1 [file PHY2-9-e14590-s001.tif]

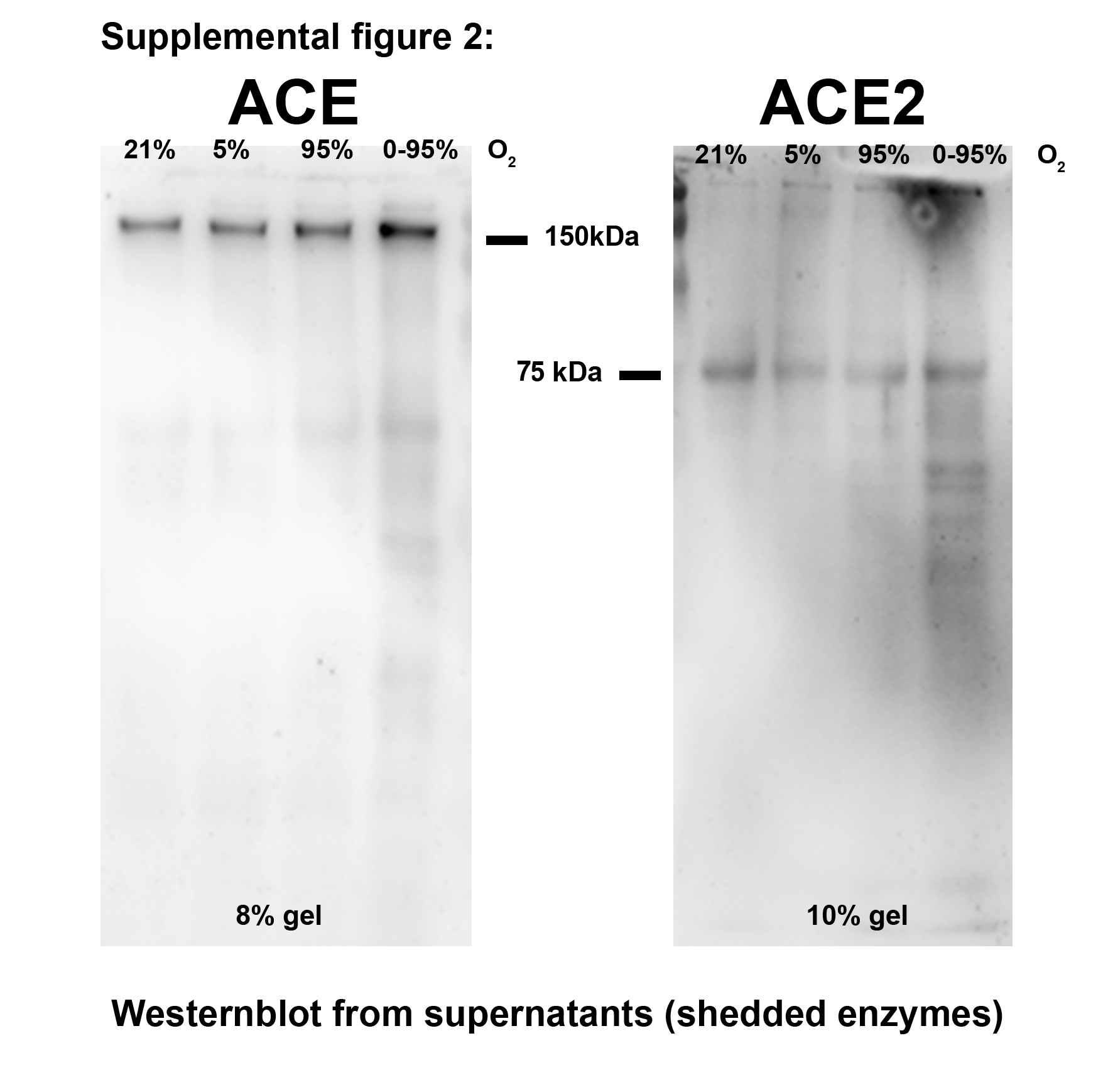

Supplement: Supplementary file 2 — Fig S2 [file PHY2-9-e14590-s002.tif]

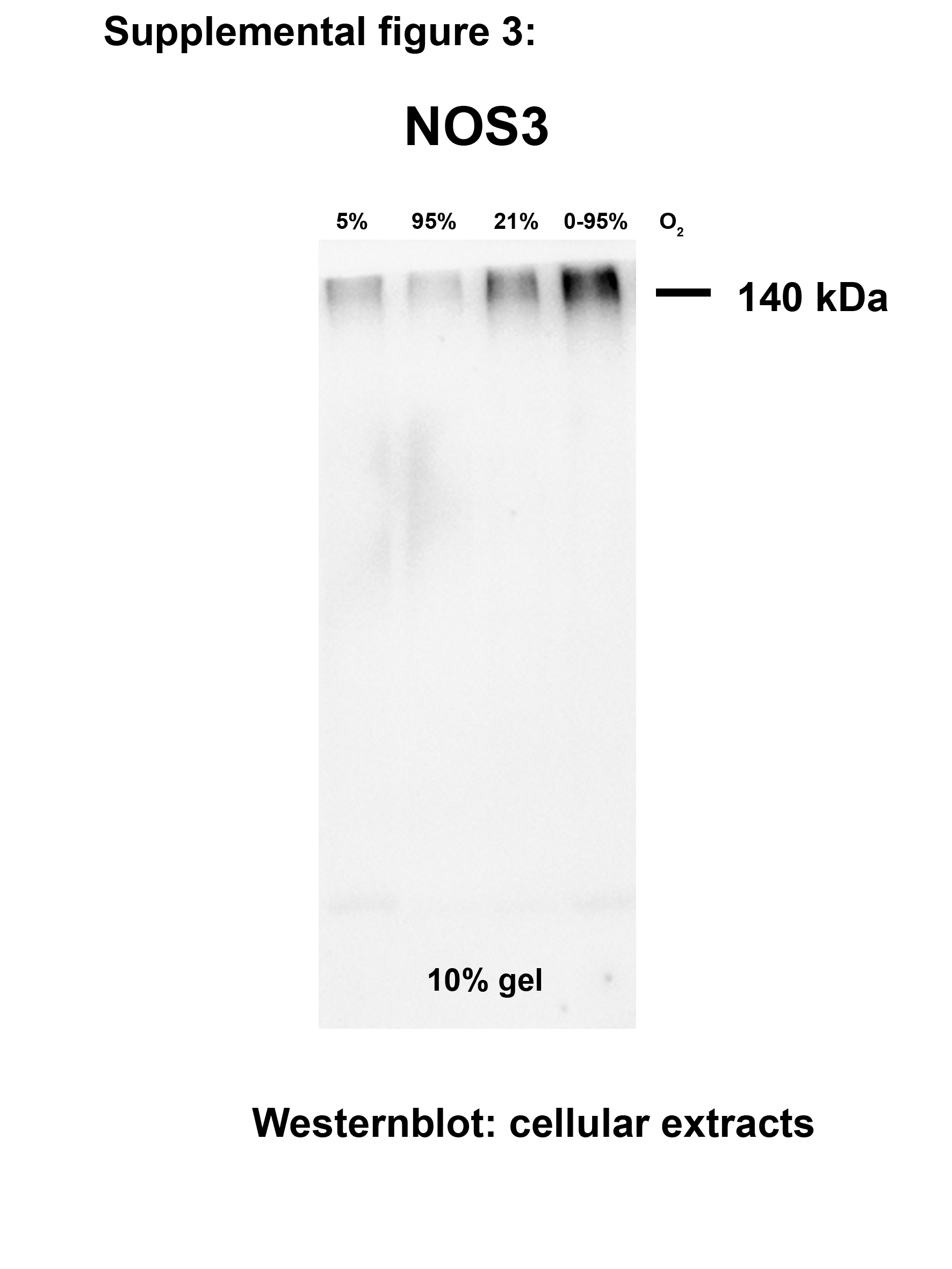

Supplement: Supplementary file 3 — Fig S3 [file PHY2-9-e14590-s003.tif]
